# Differential Responses by Human Macrophages to Infection With *Mycobacterium tuberculosis* and Non-tuberculous Mycobacteria

**DOI:** 10.3389/fmicb.2020.00116

**Published:** 2020-02-07

**Authors:** Zhihong Feng, Xiyuan Bai, Tao Wang, Cindy Garcia, An Bai, Li Li, Jennifer R. Honda, Xiuhong Nie, Edward D. Chan

**Affiliations:** ^1^Department of Respiratory Medcine, Xuanwu Hospital, Capital Medical University, Beijing, China; ^2^National Jewish Health, Denver, CO, United States; ^3^Rocky Mountain Regional Veterans Affairs Medical Center, Aurora, CO, United States; ^4^Department of Medicine, University of Colorado Anschutz Medical Campus, Aurora, CO, United States; ^5^Department of Emergency Medicine, Beijing Chaoyang Hospital, Capital Medical University, Beijing, China

**Keywords:** non-tuberculous mycobacteria, *Mycobacterium tuberculosis*, apoptosis, autophagy, nuclear factor-kappa B, cytokines

## Abstract

*Mycobacterium tuberculosis* (*MTB*) and non-tuberculous mycobacteria (NTM) are formidable causes of lung diseases throughout the world. While *MTB* is considered to be more virulent than NTM, host factors also play a key role in disease development. To elucidate whether there are differential immune responses to various mycobacteria, THP-1 macrophages were temporally infected with *MTB* H37Rv or with four different NTM species. We found that cells infected with *MTB* had greater bacterial burden and p65 nuclear factor-kappa B (NF-κB) activation than cells infected with NTM. There was also differential expression of mRNA for interleukin-1-β (IL-1β), IL-8, IL-10, and tumor necrosis factor-alpha (TNF-α) with no distinct pattern of mRNA expression among the different mycobacteria. In contrast, at the protein level, some generalizations can be made of the cytokines and chemokines expressed. Compared to uninfected cells, the rapid-growing *Mycobacterium smegmatis* but not *Mycobacterium abscessus* induced significantly greater pro-inflammatory cytokines and IL-10, whereas both NTM individually induced greater levels of chemokines. Compared to uninfected control cells, the two slow-growing NTM and *MTB* differentially induced cytokine expression with *Mycobacterium avium* inducing more pro-inflammatory cytokines and IL-10, whereas *M. avium*, *Mycobacterium intracellulare*, and *MTB* inducing greater but similar levels of chemokines. *MTB*-infected THP-1 cells also demonstrated lower level of phagosome–lysosome fusion and apoptosis than NTM-infected cells while there were differences in these macrophage functions among the NTM species. Interestingly, *M. intracellulare, M. avium*, and *MTB* have similar levels of autophagosome formation, but the levels displayed by all three were lower than for *M. smegmatis* and *M. abscessus*. This study demonstrates the differences in bacterial burden and macrophage effector functions among several clinically relevant mycobacterial species. Such disparities may, in part, account for differences in clinical outcomes among patients infected with various species of NTM as has been seen for different strains of *MTB*.

## Introduction

In the past two decades, the prevalence of non-tuberculous mycobacterial lung disease (NTM-LD) has been increasing in the United States and several parts of the world (Daniel-Wayman et al., 2019; Wagner et al., 2019). Among the ∼200 different NTM species identified, pulmonary infections are caused by relatively few, mostly due to those within the SGM group known as *Mycobacterium avium* complex (MAC) and the RGM group known as *Mycobacterium abscessus* complex. Due to the paucity of truly effective antibiotic regimens for most of the clinically relevant NTM and the specter of recurrent infections, it is difficult to achieve long-lasting cure for NTM-LD (Philley et al., 2016; Holt and Daley, 2019; Philley and Griffith, 2019).

Following inhalation or aspiration of NTM into the lower airways, the first cells encountered are most likely the airway epithelial cells, macrophages, and dendritic cells, with all three cell types capable of presenting bacterial antigens on class II MHC molecules and activating the adaptive immune response (Wosen et al., 2018). Known macrophage effector mechanisms that kill or inhibit growth of ingested mycobacteria include fusion of phagosomes and autophagosomes to lysosomes and possibly induction of apoptosis (Deretic et al., 2006; Yuk et al., 2012; Bai et al., 2013). Apoptosis of *Mycobacterium tuberculosis* (*MTB*)-infected macrophages have been associated with either favoring the host through the killing of intracellular bacteria (Keane et al., 1997; Rojas et al., 1997; Keane et al., 2002) or benefiting *MTB via* their escape from the dying cells to infect neighboring cells (Aguilo et al., 2013; Augenstreich et al., 2017). By similar mechanisms, both salubrious (Fratazzi et al., 1997; Bai et al., 2011b) and deleterious (Early et al., 2011; Bermudez et al., 2015; Whang et al., 2017) effect of apoptosis to host cells have been implicated with NTM infection. Compared to NTM, *MTB* is considered to possess greater virulence due to its increased ability to subvert the host immune response. But characterization of *MTB* with various species of NTM in the context of macrophage infection and the analysis of their effector functions are incomplete. Previous studies have investigated bacterial burden, biofilm formation by RGM (but not *M. abscessus*), apoptotic and phagosome–lysosome markers among NTM, macrophage monolayer disruption, and production of reactive oxygen intermediates (Helguera-Repetto et al., 2014; Sousa et al., 2015, 2019). In this study, we compared bacterial burden in macrophages infected with a laboratory strain of *MTB* with various SGM and RGM species and correlated this marker with some of the known effector mechanisms of killing by macrophages. While *MTB* is a slow-growing organism, SGM is used to denote only slow-growing NTM in this study.

## Materials and Methods

### Materials

The mycobacteria strains used this study were either clinical or environmental strains obtained from both National Jewish Health and American Type Culture Collection (ATCC, Manassas, VA, United States): *Mycobacterium smegmatis* mc^2^ 155, *Mycobacterium abscessus* ATCC 19977, *Mycobacterium intracellulare* NJH9141, *Mycobacterium avium* NJH87, and *MTB* H37Rv. All species were grown to log phase at 33 or 37°C in Difco Middlebrook 7H9 Medium (Becton Dickinson, MD, United States), supplemented with 10% ADC Enrichment (Remel, Lenexa, KS, United States), followed by preparation of glycerol stocks that were stored at −80°C (Bai et al., 2015a).

THP-1 cells, a human monocytic cell line, was obtained from ATCC. Reagents for Middlebrook 7H10 solid agar medium and 7H9 liquid medium were from Difco (Detroit, MI, United States), and phorbol myristate acetate (PMA) was purchased from Sigma (St. Louis, MO, United States). RPMI 1640 cell culture medium was purchased from Cambrex (East Rutherford, NJ, United States). Fetal bovine serum was from Atlanta Biologicals (Norcross, GA, United States) and heat inactivated at 56°C. Penicillin/streptomycin, LysoTracker Red DND-99, Cy3-goat anti-rabbit IgG (H + L), Lab-Tek II Chamber Slide System, NE-PER Nuclear and Cytoplasmic Extraction Reagent, and Annexin-V Human ELISA Kit were purchased from Thermo Fisher Scientific/Life Technologies (Carlsbad, CA, United States). Polyclonal rabbit anti-human LC3B, anti-p62, anti-cytochrome C, anti-Bax, anti-Bak, and β-actin antibodies, and Phototope-HRP Western Blot Detection System were purchased from Cell Signaling Technology (Danvers, MA, United States). The TransAM^®^ NF-κB p65 kit was purchased form Active Motif (Carlsbad, CA, United States). Terminal deoxynucleotidyl transferase dUTP nick end labeling (TUNEL) was determined using the *in situ* Cell Death Detection Kit, Fluorescein (Roche).

### Cell Culture and Differentiated Macrophages

THP-1 cells were cultured in RPMI-1640 medium containing 10% FBS and penicillin/streptomycin at 37°C, 5% CO_2_ with 15 ng/mL of PMA overnight. After washing twice with wash buffer (1:1 of RPMI 1640 medium:PBS), new culture medium without antibiotic was added.

### Quantitation of Cell-Associated Mycobacterial Species

In all experiments involving mycobacterial infections, THP-1 cells were infected with *MTB* and NTM species at a MOI of 10 mycobacteria:1 macrophage. In experiments that have an uninfected (control) condition (e.g., p65-NF-κB assay), an aliquot of 7H9 liquid medium – present in stock mycobacterial cultures – was added to the THP-1 cell cultures to control for any potential effects the 7H9 medium may have in the assays. After 1 h, 2 and 4 days of infection, the cells were washed, lysed, and the lysates serially diluted and plated on 7H10 solid medium to quantify cell-associated *M. smegmatis*, *M. abscessus*, *M. intracellulare*, *M. avium*, and *MTB* H37Rv. GFP-labeled *Mycobacterium* species were generated as previously described (Bai et al., 2013).

### p65 NF-κB Binding Assay

Activation and binding of the p65 subunit of NF-κB to its *cis*-regulatory element was quantified. For each condition, 10 μg nuclear protein extract from cells was prepared and incubated in 96-well plates coated with an oligonucleotide containing the NF-κB consensus binding site (5′-GGGACTTTCC-3′ oligonucleotide) for 1 h at room temperature. In addition, soluble wildtype and mutated consensus oligonucleotides were used as a specific competitor and non-competitor, respectively, for p65-NF-κB binding. After washing three times, NF-κB p65 antibody was added for 2 h following by HRP-conjugated secondary antibody, binding of activated p65 NF-κB was determined colorimetrically, all according to manufacturer’s instructions.

### RNA Isolation and qRT-PCR

THP-1 cells were plated onto six-well tissue culture plates seeded with 1 × 10^6^ cells per well. Total RNA was extracted using the Trizol reagent, and cDNA was prepared using High-Capacity cDNA Reverse Transcription Kit, according to the manufacturer’s instructions (Applied Biosystems). qPCR was performed by using SYBR Green PCR Master Mix in Applied Biosystems qPCR Assays instrument. The following primers were used: interleukin-1-beta (IL-1β) F: 5′-CAGCTACGAATC TCCGACCAC-3′, R: 5′-GGCAGGGAAC CAGCATCTTC-3′; interleukin-8 (IL-8) F: 5′-ACTGAGAGTGA TTGAGAGTGGAC-3′, R: 5′-AACCCTCTG CACCCAGTTT TC-3′; tumor necrosis factor-alpha (TNF-α) F: 5′-CCTCTCTCT AATCAGCCCTCTG-3′, R: 5′-GAGGACCTGGGAGTAGATG AG-3′; interleukin-10 (IL-10) F: 5′-CAACCTGCCTAACATGC TTCG-3′, R: 5′-TCATCTCAGACAAGGCT TGGC-3′; GAPDH F: 5′-AGGGGAGATTCA GTGTGGTG3′, R: 5′-AGGGGAGA TTCAGTGTGGTG-3′.

### Cytokine Analysis

Supernatants of differentiated THP-1 macrophages infected with different mycobacterial species for 1 h, 2, and 4 days were quantified for lL-1β, IL-6, lL-8, lL-10, lL-12p40, IL-12p70, monocyte chemoattractant protein-1 (MCP-1), macrophage inflammatory protein-1-alpha (MIP1α), and TNF-α using HCYTOMAG-60K/MILLIPLEX^®^ MAP Human Cytokine/Chemokine Magnetic Bead Panel-Immunology Multiplex Assay (EMDMILLIPORE Inc., Temecula, CA, United States) with the Luminex MAGPIX instrument (Luminex Inc.).

### Quantitation of Phagosome–Lysosome Fusion, Autophagosome Formation, and Apoptosis

In brief, THP-1 cells were seeded on glass chamber slides, cultured in RPMI medium with 15 ng/mL PMA overnight, washed and replaced with culture medium without PMA, and infected with GFP-*M. smegmatis*, GFP-*M. abscessus*, GFP-*M. intracellulare*, GFP-*M. avium*, or GFP-*MTB* H37Rv at a MOI of 10:1 for various time points. Phagosome–lysosome fusion (LysoTracker Red DND-99), autophagosome formation (immunofluorescent staining with rabbit anti-LC3 antibody and Cy3-tagged anti-rabbit antibody), and apoptosis (TUNEL) and expression of annexin-V, cytochrome C, Bax, and Bak were analyzed as previously described (Bai et al., 2010, 2013, 2015b).

### Western Blotting

Differentiated THP-1 cells were lysed with a cell lysis buffer, and protein concentration of cell lysates was determined using the Bradford protein assay (Bio-Rad) (Chan et al., 1997). Thirty micrograms of protein for each condition was resolved by 12% SDS–PAGE and transferred onto iBLOT PVDF membrane using transfer machine from Invitrogen (Thermo Fisher Scientific). After the membrane were blocked in 5% of dry milk for 1 h, they were probed with cytochrome C, Bax, Bak, or β-actin antibodies at a dilution of 1:1000 (v/v), followed by detection with HRP-conjugated anti-rabbit IgG (1:2000 dilution). The bands were visualized by using of SuperSignal West Femto Maximum Sensitivity Substrate System (Thermo Fisher Scientific).

### Statistical Analysis

Data are presented as the mean ± SEM of three independent experiments, unless otherwise indicated. Group means were compared by repeated-measures ANOVA using Fisher’s least significant test or by two-way ANOVA with Bonferroni’s *post hoc* test.

## Results

### Differential Burden of Mycobacteria in Infected Macrophages

**FIGURE 1:**
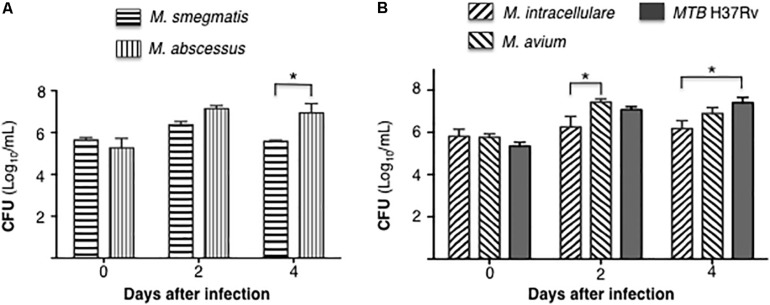
Differential burden mycobacteria in human THP-1 macrophages. **(A)** Quantitation of cell-associated *M. smegmatis* and *M. abscessus* following 1 h, 2 and 4 days after infection of THP-1 macrophages (CFU log_10_) using a multiplicity-of-infection (MOI) of 10 mycobacteria:1 macrophage. **(B)** Quantitation of cell-associated *M. intracellulare, M. avium*, and *MTB* H37Rv following 1 h, 2 and 4 days after infection of THP-1 macrophages (CFU log_10_). Data shown are the mean ± SEM of three independent experiments. *p < 0.05.

Differentiated THP-1 macrophages were infected with each of the five mycobacterial species at a MOI of 10 mycobacteria:1 macrophage. CFU were enumerated at 1 h, 2 and 4 days after infection. Comparing the RGM, CFU counts of *M. abscessus* were significantly higher than *M. smegmatis* at 4 days after infection (Figure 1A). With the SGM and *MTB*, the cell-associated burden of *M. avium* was significantly more than *M. intracellulare* at 2 days after infection, while the bacterial burden of *MTB* H37Rv was significantly more than *M. intracellulare* at 4 days after infection (Figure 1B).

### *MTB* H37Rv Induces More p65-NF-κB Activation and Differential Cytokine Expression Compared to NTM in Human Macrophages

**FIGURE 2:**
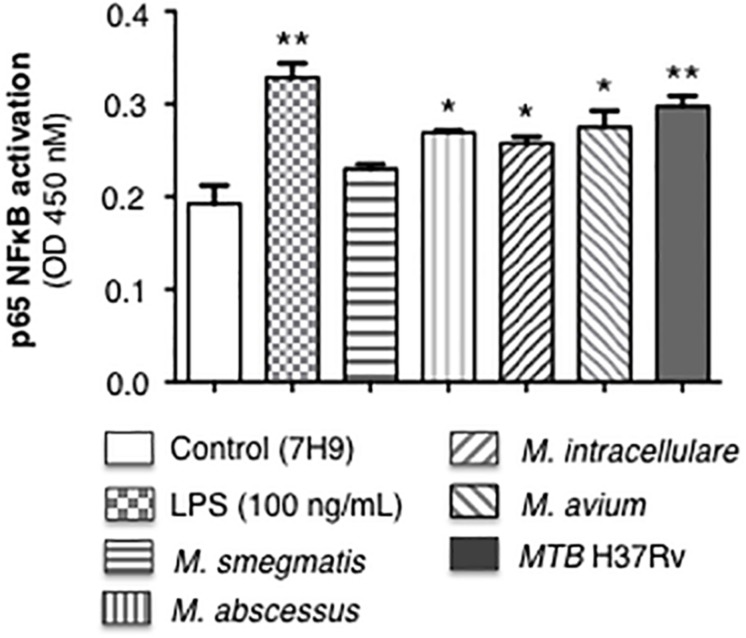
p65-NF-κB activation and binding in response to infection of THP-1 macrophages with different mycobacterial species. THP-1 cells were stimulated with LPS (positive control) or infected with the mycobacterial species at a multiplicity-of-infection (MOI) of 10 mycobacteria:1 macrophage for 6 h, nuclear extracts prepared, and p65-NF-κB binding to its consensus *cis*-regulatory element was quantified by Active Motif TransAM^TM^ Flexi. Data shown are mean ± SEM of three independent experiments. *p < 0.05, **p < 0.01 compared to uninfected control cells, to which a small volume of 7H9 medium was added, equal in volume to the stock mycobacteria (grown in 7H9 medium) used to inoculate the THP-1 cells.

Mycobacteria initiate immune cell activation, in part, by engagement of specific mycobacterial ligands with pattern-recognition receptors such as the toll-like receptors. One consequence of these interactions is NF-κB activation and induction of chemokines and cytokines that recruit or activate other immune cells. We previously reported that *MTB* infection of human macrophages induced NF-κB activation, which then inhibited autophagosome formation (Bai et al., 2013). Differentiated THP-1 macrophages were infected with the individual mycobacterial species or stimulated with lipopolysaccharide (positive control) for 6 h, followed by quantitation of p65-NF-κB binding to its consensus oligonucleotide sequence. *MTB* H37Rv induced the most p65-NF-κB activation and binding whereas avirulent *M. smegmatis* induced the least (Figure 2).

**FIGURE 3:**
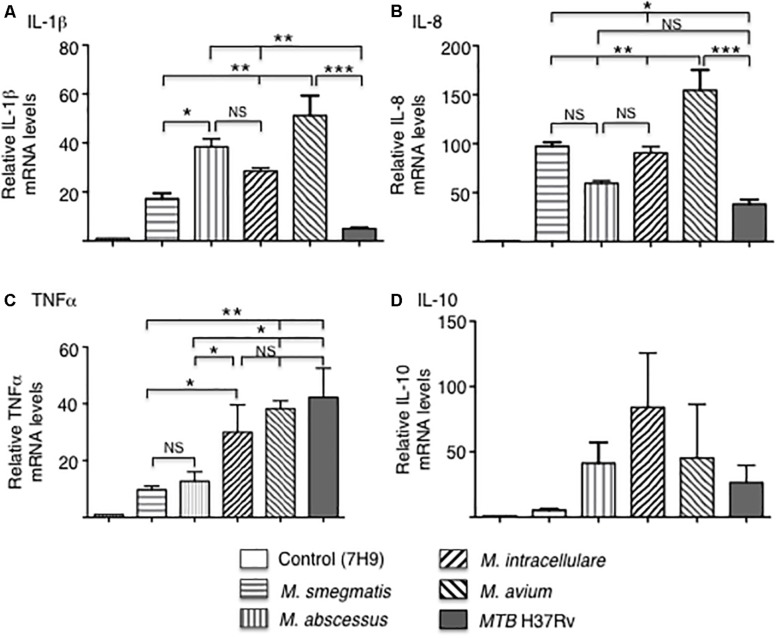
Cytokine gene expression by THP-1 cells infected with different mycobacterial species. Quantitative mRNA expression of **(A)** IL-1β, **(B)** IL-8, **(C)** TNF-α, and **(D)** IL-10 were determined by qRT-PCR after infection of the indicated mycobacteria for 18 h. GAPDH was used as an endogenous control in the comparative ΔΔCt method. Data shown are mean ± SEM of three independent experiments. *p < 0.05, **p < 0.01, ***p < 0.001, NS, not significant. MOI of 10 mycobacteria:1 macrophage.

The mRNA expression of the pro-inflammatory cytokines IL-1β, IL-8, and TNF-α, as well as the anti-inflammatory cytokine IL-10 were quantified by qRT-PCR after infection of THP-1 macrophages with the various mycobacteria for 18 h. In *MTB*-infected macrophages, IL-1β and IL-8 mRNA expression was the lowest whereas TNF-α mRNA expression level was the highest. Among the NTM, *M. avium*-infected cells produced the greatest mRNA levels of IL-1β, IL-8, and TNF-α while the RGM generally induced lower levels of cytokines with *M. smegmatis* inducing the least amount of IL-1β, TNF-α, and IL-10 (Figure 3). There was no significant difference in IL-10 levels in macrophages infected with different mycobacteria species although *M. smegmatis* and *MTB* produced lower amounts. However, cytokine and chemokine protein expression do not always correlate with mRNA expression, even qualitatively, due to a number of factors including mRNA stability and complex translational regulation (Bai et al., 2011a). Hence, we also measured secreted cytokine and chemokine levels.

**FIGURE 4:**
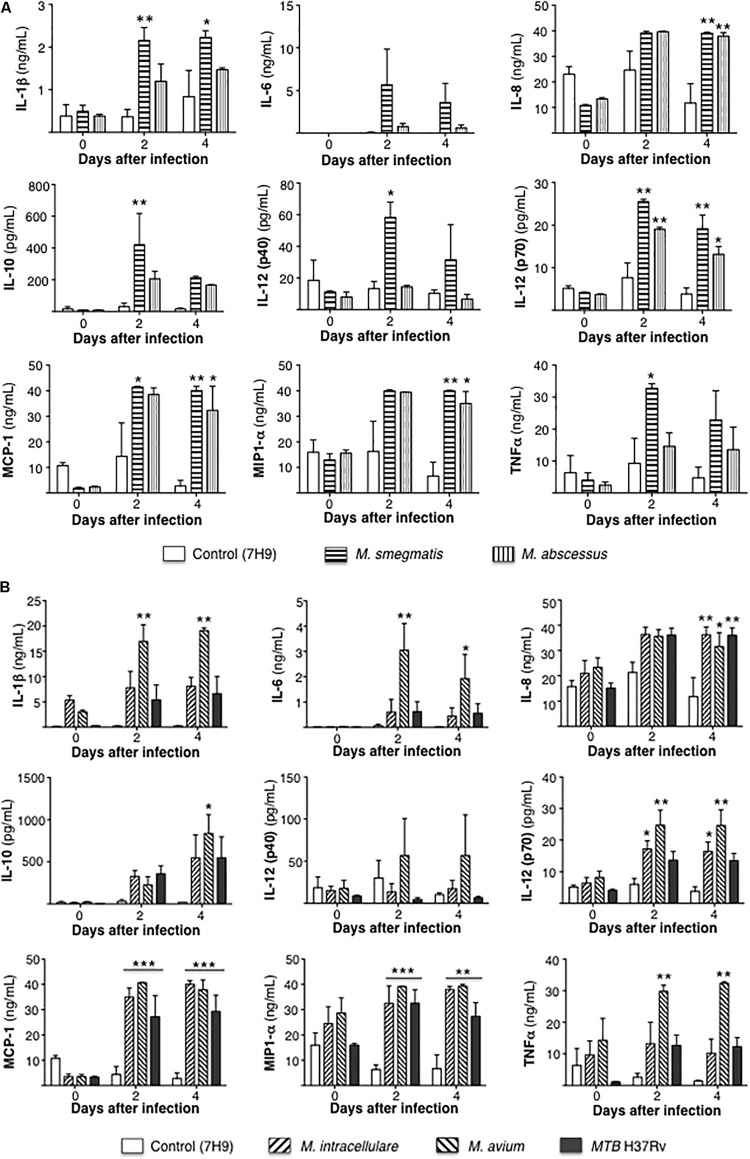
Differential cytokine expression by mycobacterial species. **(A)** Cytokine expression 1 h, 2 and 4 days after infection of THP-1 cells with rapid-growing NTM (*M. smegmatis* and *M. abscessus*). **(B)** Cytokine expression 1 h, 2 and 4 days after infection of THP-1 cells with slow-growing NTM (*M. intracellulare* and *M. avium*) and *MTB*. Data shown are the mean ± SEM of three independent experiments. *p < 0.05, **p < 0.01, ***p < 0.001 compared to respective control (open) bar. Multiplicity-of-infection of 10 mycobacteria:1 macrophage.

Secreted cytokine protein levels were quantified in the culture supernatant at 1 h, 2 and 4 days after infection. To simplify the comparisons, cytokine measurements for the RGM-infected THP-1 cells are reported separately from the cells infected with SGM or *MTB*. For the RGM, compared to uninfected THP-1 cells, the less virulent *M. smegmatis* but not *M. abscessus* induced greater production of the pro-inflammatory cytokines IL-1β, IL-12p40, and TNF-α but also more IL-10. But both RGM induced greater levels of IL-8, IL-12p70, MCP-1, and MIP1α compared to uninfected cells (Figure 4A).

Compared to uninfected controls, only *M. avium*-infected cells produced greater levels of IL-1β, IL-6, TNF-α, and IL-10 whereas *M. intracellulare* and *M. avium* induced greater levels of IL-12p70, and *M. intracellulare*, *M. avium*, and *MTB* induced more IL-8, MCP-1, and MIP1α (Figure 4B).

### Differential Phagosome–Lysosome Fusion, Autophagosome Formation, and Apoptosis Among Mycobacteria-Infected Macrophages

**FIGURE 5:**
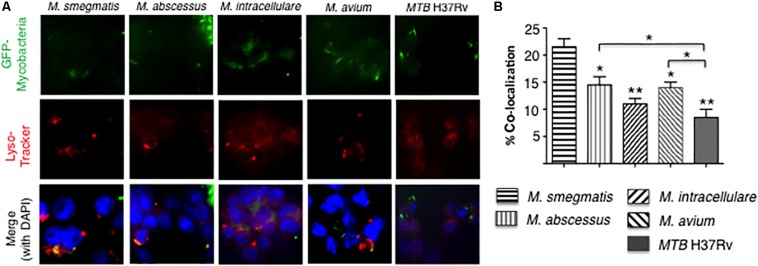
More virulent mycobacteria induced less phagosome–lysosome fusion in macrophages. **(A)** Localization of phagosome–lysosome fusion in human THP-1 macrophages following infection with GFP-labeled mycobacteria for 6 h. Shown are representative fluorescent photomicrographs of three independent experiments. **(B)** Quantification of the percentage of THP-1 cells with co-localization of GFP-labeled mycobacteria and lysosomes. Data shown are the mean ± SEM of three independent experiments. Unless otherwise indicated, the individual *p < 0.05 and **p < 0.01 are compared to *M. smegmatis*-infected cells. Multiplicity-of-infection of 10 mycobacteria:1 macrophage.

To quantify phagosome–lysosome fusion in the mycobacteria-infected macrophages, THP-1 cells were infected with GFP-labeled mycobacteria and incubated with LysoTracker Red (Figure 5A). The percentage of cells with co-localization of GFP-labeled mycobacteria and lysosomes was determined. *M. smegmatis*-infected cells have significantly greater number of phagosome–lysosome fusion compared to all other mycobacteria-infected cells. In addition, *MTB* H37Rv-infected THP-1 cells also have significantly fewer number of phagosome–lysosome fusion compared to either *M. abscessus*- and *M. avium*-infected cells (Figure 5B).

**FIGURE 6:**
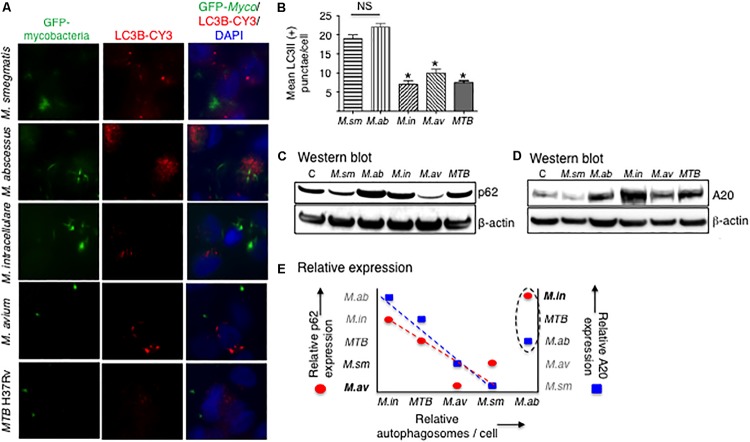
More virulent mycobacterial species generally induced less autophagosome formation. **(A)** Identification of autophagosomes by immunofluorescent detection of LC3II-positive particles 24 h after infection of THP-1 cells by the different mycobacteria. Shown photomicrographs are representative of three independent experiments. **(B)** Quantification of the number of autophagosomes, reported as the average number of autophagosomes per cell. Data represent the mean ± SEM of three independent experiments. **(C)** Immunoblot for p62 expression of THP-1 cells infected for 24 h with the mycobacterial species. **(D)** Immunoblot for A20 expression of THP-1 cells infected for 24 h with the mycobacterial species. Both immunoblots shown are representative of three independent experiments. **(E)** Qualitative graphical representation of the relative expression of autophagosome number as a function of p62 (red circles) and A20 (blue squares) expression. The dashed lines are an estimate of the lines of best fit, excluding points for *M. abscessus*. *M. sm*, *M. smegmatis*; *M. ab*, *M. abscessus*; *M. in*, *M. intracellulare*; *M. av*, *M. avium*; *MTB*, *M. tuberculosis*. *p < 0.05 compared to *M. smegmatis*. Multiplicity-of-infection of 10 mycobacteria:1 macrophage.

Increase in LC3II and a decrease in p62 expression are two important indicators in autophagosome formation and maturation, respectively. Autophagosome formation in THP-1 cells infected with the different mycobacterial species was quantified by immunofluorescence of LC3-II positive autophagosomes. At 24 h after infection with the different mycobacterial species, autophagosome numbers were similar between the two RGM and similar among SGM and *MTB* although the RGM-infected macrophages have greater number of autophagosomes than SGM- or *MTB*-infected macrophages (Figures 6A,B). Compared to control uninfected cells, p62 expression at 24 h of infection showed a decrease in *M. smegmatis* and *M. avium*-infected THP-1 cells (Figure 6C), indicating at this time point, there was greater autophagosome maturation in macrophages infected with these two NTM.

Tumor necrosis factor alpha-induced protein 3 is a deubiquitinating protein that inhibits TRAF6 and thus prevents TRAF6 from ubiquitinating a key autophagic protein Beclin-1 (Vural and Kehrl, 2014). A20 protein expression was determined by immunoblotting in THP-1 cells after 24 h of infection with the different NTM species and *MTB*. *M. intracellulare* and *MTB* induced the greatest amounts of A20, followed by *M. abscessus* and *M. avium*, and least amount of A20 was induced by *M. smegmatis* (Figure 6D).

To qualitatively compare the different mycobacterial species in terms of the protein markers that are involved in the autophagic process, we graphically analyzed their relative expression of autophagosome number, p62 expression, and A20 expression at a single time point of 24 h. As shown in Figure 6E, *M. abscessus* is an outlier in the relationship between these three variables. Thus, excluding the data for *M. abscessus*, autophagosome number is inversely related to A20 expression (Figure 6E, closed blue squares), consistent with the aforementioned function of A20. However, the inverse relationship between p62 and autophagosome number (Figure 6E, closed red circles) is more difficult to explain – see the section “Discussion” – as one would normally expect a direct relationship between p62 expression and autophagosome number. Nevertheless, even if the data for *M. abscessus* were included in the qualitative analysis, similar trends were also seen albeit not as robust.

**FIGURE 7:**
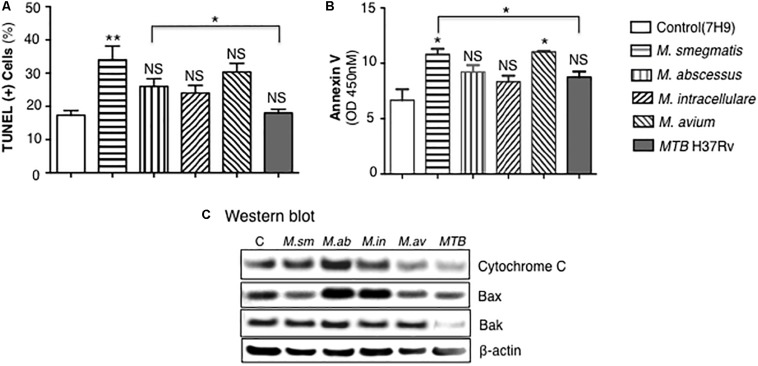
*MTB* H37Rv induced less apoptosis than other mycobacterial species. **(A)** Quantitation of THP-1 cell apoptosis by TUNEL staining 24 h after infection with the individual mycobacterial species. **(B)** Annexin-V expression was quantified in THP-1 macrophages infected overnight with various mycobacterial species. Data shown are the mean ± SEM of three independent experiments. **(C)** Cytochrome C, Bax, and Bak expression was determined by immunoblotting of the lysates of THP-1 cells infected with various NTM species and *MTB* H37Rv. Immunoblots shown are representative of three independent experiments. Unless otherwise indicated, *p < 0.05, **p < 0.01 are compared to unstimulated control cells (open bars). Multiplicity-of-infection of 10 mycobacteria:1 macrophage.

Apoptosis of mycobacteria-infected THP-1 cells were quantified by TUNEL as well as by expression of annexin-V, cytochrome C, Bax, and Bak. THP-1 cells infected with mycobacteria for 24 h showed significant increase in apoptosis only for the *M. smegmatis*-infected cells when compared to uninfected cells although there was trend toward increase with the other NTM but not with *MTB* (Figure 7A). *M. smegmatis* and *M. avium* demonstrated significant increase in annexin-V expression but the other mycobacteria did not compared to uninfected cells (Figure 7B). *MTB*-infected macrophages had, in general, the lowest amount of apoptosis, albeit the differences were modest. We further studied the pro-apoptotic pathway by immunoblotting for cytochrome C, Bax, and Bak expression following 24 h of mycobacterial infection. Immunoblotting showed that the relative expression of cytochrome C was most notably increased in *M. abscessus*-infected THP-1 cells and decreased with *M. avium* or *MTB* infection (Figure 7C). Relative Bax expression was increased in cells infected with *M. abscessus* or *M. intracellulare* whereas Bak was significantly decreased in cells infected with *MTB*.

## Discussion

Understanding virulence strategies of different mycobacterial species may aid in the search for novel drug targets. In this study, we compared macrophage responses to infection with *MTB* H37Rv and different species of NTM, specifically in regards to bacterial burden, cytokine expression, phagosome–lysosome fusion, autophagosome formation and a marker for maturation, and apoptosis. While primary human macrophages are more physiologically relevant, the reason we utilized THP-1 macrophages is twofold: (i) we wished to compare different strains of mycobacteria and thus did not want to introduce confounding variables with use of primary macrophages from different donors and (ii) THP-1 cells are well-established human cells shown to be a good *in vitro* model to study mycobacterial infections and comparable to primary human macrophages (Bai et al., 2013).

**FIGURE 8:**
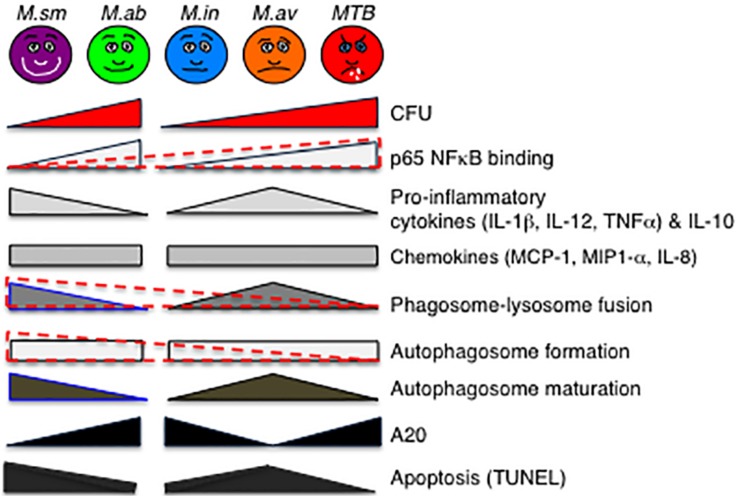
Qualitative diagram of the relationship between CFU and effector functions of macrophages infected with *MTB* and NTM. See the section “Discussion” in text. For p65-NF-κB activation, phagosome–lysosome fusion, and autophagosome formation, the data are displayed separately for the RGM (*M. smegmatis* and *M. abscessus*) and SGM (*M. intracellulare*, *M. avium*) plus *MTB* (different geometrical shapes with various shades of gray) but also combined (overlaying red-dashed triangles). While it is more difficult to generalize the relative induction of apoptosis with the NTM species, *MTB* induced the least amount of apoptosis and apoptotic phenotypic markers in the infected macrophages. CFU, colony forming units; NF-κB, nuclear factor kappa B; *M. sm*, *M. smegmatis*; *M. ab*, *M. abscessus*; *M. in*, *M. intracellulare*; *M. av*, *M. avium*; *MTB*, *M. tuberculosis*.

Given differences in proliferation rate, we compared the bacterial burden of the two RGM separately from the SGM and *MTB*. There was a trend toward greater number of *M. abscessus* at day 2 of infection and over one log_10_ greater at day 4, consistent with the avirulent nature of *M. smegmatis* (Figure 8). In comparing intracellular burden of the SGM and *MTB*, *M. intracellulare* showed the least number of bacteria at days 2 and 4 whereas *MTB* showed the most at day 4 (Figure 8). The finding that *M. intracellulare* had a trend toward lesser bacterial burden compared to *M. avium* is not consistent with clinical findings that isolation of *M. intracellulare* is more likely to be predictive of lung disease and that it is associated with more severe presentation and worse outcome (Han et al., 2005; Koh et al., 2012). However, another study found that *M. avium* cultured from clinical specimens was more likely to be associated with lung disease than when *M. intracellulare* was isolated (Stout et al., 2008). The disparities in these studies may also be due to use of different strains of MAC species by the investigators since it is well established that different strains of the same species of MAC may have divergences in virulence, as has been shown for *M. avium* subsp *hominissuis*; i.e., wherein phagocytosis of sequevars Mav-A and Mav-B – which differed in their base sequence of the 16S-23S ribosomal internal transcribed spacer (ITS) – by THP-1 cells and monocyte-derived human macrophages did not differ but by Day 5 of infection, the bacterial burden of Mav-A organisms was significantly more than the Mav-B organisms (Rindi et al., 2018).

An interesting pattern of differential cytokine and chemokine production was observed with both the RGM-infected macrophages and the SGM- or *MTB*-infected macrophages (Figure 8). In general, *M. smegmatis* induced more pro-inflammatory cytokines (IL-1β, IL-6, IL-12, and TNF-α) and IL-10 than *M. abscessus* but these two RGM induced similar amounts of the chemokines (IL-8, MCP-1, MIP1α). With the SGM and *MTB*, *M. avium* clearly induced greater production of IL-1β, IL-6, IL-12p70, TNF-α, and IL-10 whereas these three mycobacteria induced similar levels of IL-8, MCP-1, and MIP1α. While there were variations in the relative induction of the host effector functions, *MTB* H37Rv induced more p65 NF-κB activation and less phagosome–lysosome fusion, and autophagosome numbers compared to nearly all of the NTM (Figure 8). In this context, we previously showed that in THP-1 cells, human monocyte-derived macrophages, and human alveolar macrophages that p65-NF-κB activation – while important for the pro-inflammatory response – inhibits autophagosome formation (Bai et al., 2013). *M. smegmatis*-infected cells have the greatest mean phagosome–lysosome fusion among the species tested, which may account for its relative avirulence (Figure 8). While the percent co-localization of mycobacteria with lysosomes – quantified by the number of cells that demonstrate co-localization of GFP-labeled mycobacteria with lysosomes divided by the number of GFP-infected cells – was relatively low, it is important to note that infection of macrophages can be quite heterogenous; i.e., some macrophages phagocytose no mycobacteria whereas others phagocytose many. Thus, the presence of P–L fusion in macrophages that contain many NTM will be more biologically significant than those that contain fewer. Furthermore, the number of *M. smegmatis* did not decline consistently over the period of infection – belying its known avirulent nature – this may be because the *in vitro* experiments were performed in macrophages whereas *in vivo*, NTM are exposed to a myriad of other cell types acting in collaboration against the NTM.

In THP-1 cells infected with different mycobacterial species, LC3-II expression – as determined by fluorescent immunocytochemistry – showed that the SGM and *MTB* induced less autophagosomes than the RGM. While less autophagosomes seen with SGM and *MTB*-infected macrophages could also mean increased autophagosome–lysosome fusion as displayed by reduced p62 levels, this was not consistently seen with the SGM and *MTB* with the exception of *M. avium*; i.e., the relatively low number of autophagosomes in *M. avium*-infected macrophages (Figure 6B) may be due, in part, to increased autophagosome maturation as evinced by the corresponding reduced p62 level (Figure 6C). Nevertheless, these findings are consistent with the SGM- and *MTB*-infected macrophages inducing overall greater A20 expression than RGM-infected macrophages since A20 is a deubiquitinating enzyme that inhibits autophagosome formation. We also compared qualitatively the relative expression of p62, A20, and autophagosome number with the caveat that this comparison is limited by the presence of only one time point for each of the variables in all the mycobacterial species; i.e., it is likely that each distinct mycobacterial species will have different kinetics in the autophagic flux. The inverse relationship between increased A20 expression and reduced autophagosome number is entirely consistent with the inhibitory function of A20 on autophagosome formation. On the other hand, we struggled to explain the inverse relationship between p62 and autophagosome number as increased p62 expression is generally associated with either increased autophagosome formation or decreased autophagosome maturation, with the latter resulting in increased p62 due to decreased p62 degradation as a result of decreased autophagosome–lysosome fusion. We posit that one possible explanation for the inverse relationship between p62 and autophagosome number is if there were differences in the kinetics of the autophagic flux between the different mycobacterial species as our qualitative analysis is limited by the single time point examined for all the mycobacterial species.

The role apoptosis plays in NTM-infected macrophages is controversial since both host-protection (Fratazzi et al., 1997; Bai et al., 2011b) and NTM survival (Early et al., 2011; Bermudez et al., 2015; Whang et al., 2017) have been implicated with apoptosis. Compared to *M. smegmatis* and other NTM, *MTB* H37Rv induced the least amount of apoptosis as evinced by lower number of TUNEL-positive cells as well as lower expression of annexin-V, cytochrome C, Bax, and Bak. Interestingly, cells infected with the least virulent *M. smegmatis* displayed the most TUNEL-positive cells whereas *M. abscessus* and *M. intracellulare* induced the most cytochrome C, Bax, and perhaps Bak expression. Thus, while it is more difficult to correlate induction of apoptotic markers with NTM virulence, one fairly consistent finding was that *MTB* – considered the most virulent among the mycobacteria examined – induced the least amount of apoptosis of the macrophages as well as the least induction of apoptotic phenotypic markers.

Others have also compared various species of NTM with or without comparison to *MTB* in macrophage models of infection (Helguera-Repetto et al., 2014; Sousa et al., 2015, 2019). While mycobacterial burden was consistently quantified in these studies, significant differences in the times of infection and in the assays analyzed make these studies and ours complementary. Sousa et al. (2015) investigated the ability of three RGM (*M. smegmatis, M. fortuitum*, and *M. chelonae*) to replicate in THP-1 cells and to form biofilms. Infection and quantitation of intracellular NTM demonstrated that the bacterial burden in macrophages occurred in the following order: *M. fortuitum* > *M. chelonae* > *M. smegmatis*. They also found that, overall, *M. smegmatis* and *M. fortuitum* were better biofilm formers than *M. chelonae* (Sousa et al., 2015). In a subsequent study from the same group, infection with *M. smegmatis, M. fortuitum* (clinical and reference strains), and *M. avium* (clinical and reference strains) were compared in THP-1 cells (Sousa et al., 2019). They found that *M. smegmatis* and reference strain of *M. fortuitum* were cleared relatively quickly in the macrophages whereas both strains of *M. avium* and the clinical strain of *M. fortuitum* were able to replicate. Although phagosome and lysosome markers were quantified in the cells, co-localization of mycobacteria with lysosomes was not. Caspase 8 and caspase 3/7 positive cells were quantified, showing that these caspases were induced more by *M. fortuitum* and *M. avium* up to 6 h; however, after 1 day, *M. smegmatis* and *M. fortuitum* induced greater caspase 3/7 positive cells (Sousa et al., 2019). But quantitation of actual apoptotic cells (*e.g.*, TUNEL staining) was not performed. Helguera-Repetto et al. (2014) compared THP-1 cells infected with *M. abscessus*, *M. fortuitum*, *M. celatum*, and *MTB*. They found that over a two day period of infection, there was increased growth of *M. abscessus* and *M. fortuitum* but the number of *M. celatum* and *MTB* remain relatively constant temporally (Helguera-Repetto et al., 2014). The significant differences in the experiments conducted between their study and ours – including differences in MOI used, times of infection before washing the cell culture, maximum time of infection, NTM species used, and macrophage effector function assayed – make these two studies complementary.

Two additional *in vitro* studies compared cytokine expression of various mycobacteria in macrophages (Beltan et al., 2000; Song et al., 2003). Beltan et al. (2000) infected human monocyte-derived macrophages with various pathogenic mycobacteria (*MTB*, *M. avium*, *M. kansasii*, or *M. xenopi*) and non-pathogenic mycobacteria (*M. smegmatis*, *M. phlei*) and measured induction of various cytokines. They found that the non-pathogenic NTM induced greater levels of TNF-α and granulocyte monocyte-colony stimulating factor and, to a lesser extent, more IL-6 than the pathogenic mycobacteria (Beltan et al., 2000). Our findings were more variable in that *M. smegmatis* and *M. avium* induced similar but more TNF-α compared to *M. abscessus*, *M. intracellulare*, or *MTB*. Song et al. (2003) measured IL-8 levels in human monocytes and showed that *M. smegmatis* induced significantly greater levels of IL-8 than cells infected with *MTB* H37Rv. While we also saw more IL-8 with *M. smegmatis* infection compared to *MTB* infection, the difference was modest and insignificant. One possible reason for the discrepancy between the study by Song et al. (2003) and ours is that different monocytic cell lines were used; i.e., U937 cells in their study vs. THP-1 cells in ours.

Similar in concept, two studies investigated whether there were differences in virulence in NTM that caused nodular–bronchiectasis vs. cavitary disease (Sohn et al., 2009; Tatano et al., 2010). Tatano et al. (2010) compared the infection of *M. avium* strains isolated from those with nodular-bronchiectasis (generally considered milder form of disease) vs. cavitary lung disease (generally considered more severe) in various types of human cells and found no difference in their ability to replicate or produce reactive nitrogen or oxygen intermediates. In contrast, Sohn et al. (2009) found that *M. abscessus* isolated from those with cavitary NTM lung disease were able to replicate faster and induced higher levels of cytokines compared to *M. abscessus* strains isolated from those with nodular-bronchiectasis NTM lung disease, indicating lesser *M. abscessus* virulence in the latter cases.

There are several limitations to this study. One is that THP-1 cells were used rather than primary human macrophages; but the main reason we employed THP-1 cells was that in comparing the different mycobacteria, we did not want the macrophages to be a variable – which would not be the case if primary macrophages were used from different donors or even from the same donor obtained at different times. Another limitation is that autophagic flux was not examined in a temporal fashion with the different mycobacterial species.

## Conclusion

In conclusion, this study revealed differences in host macrophage–pathogen interactions among several mycobacterial species, adding to our understanding of the mechanisms by which NTM cause sustained infection of macrophages. Further elucidation of the host–NTM interaction in macrophages and other cell types may ultimately lead to identifying more novel drug targets for which, overall, these infections have a poor response to treatment.

## Data Availability Statement

The raw data supporting the conclusions of this article will be made available by the authors, without undue reservation, to any qualified researcher.

## Author Contributions

ZF, XB, and EC conceptualized and designed the study, and prepared the first draft and sections of the manuscript. ZF, XB, TW, CG, AB, LL, JH, XN, and EC worked on the analysis and interpretation, read, reviewed, and approved the manuscript.

## Conflict of Interest

The authors declare that the research was conducted in the absence of any commercial or financial relationships that could be construed as a potential conflict of interest.

## Abbreviations

A20: tumor necrosis factor alpha-induced protein 3
CFU: colony forming units
LC3: light chain 3
MOI: multiplicity-of-infection
NF- κ B: nuclear factor-kappa B
NTM: non-tuberculous mycobacteria
RGM: rapid-growing mycobacteria
SGM: slow-growing mycobacteria
TB: *tuberculosis*.
